# Structure-based directed evolution improves *S. cerevisiae* growth on xylose by influencing in vivo enzyme performance

**DOI:** 10.1186/s13068-019-1643-0

**Published:** 2020-01-11

**Authors:** Misun Lee, Henriëtte J. Rozeboom, Eline Keuning, Paul de Waal, Dick B. Janssen

**Affiliations:** 1 Biochemical Laboratory, Groningen Biomolecular Sciences and Biotechnology Institute, University of Groningen, Nijenborgh 4, 9747 AG Groningen, The Netherlands; 2 DSM Biotechnology Center, Alexander Fleminglaan 1, 2613 AX Delft, The Netherlands

**Keywords:** Xylose isomerase, Metalloenzyme, Directed evolution, Bioethanol, Protein engineering

## Abstract

**Background:**

Efficient bioethanol production from hemicellulose feedstocks by *Saccharomyces cerevisiae* requires xylose utilization. Whereas *S. cerevisiae* does not metabolize xylose, engineered strains that express xylose isomerase can metabolize xylose by converting it to xylulose. For this, the type II xylose isomerase from *Piromyces* (PirXI) is used but the in vivo activity is rather low and very high levels of the enzyme are needed for xylose metabolism. In this study, we explore the use of protein engineering and in vivo selection to improve the performance of PirXI. Recently solved crystal structures were used to focus mutagenesis efforts.

**Results:**

We constructed focused mutant libraries of *Piromyces* xylose isomerase by substitution of second shell residues around the substrate- and metal-binding sites. Following library transfer to *S. cerevisiae* and selection for enhanced xylose-supported growth under aerobic and anaerobic conditions, two novel xylose isomerase mutants were obtained, which were purified and subjected to biochemical and structural analysis. Apart from a small difference in response to metal availability, neither the new mutants nor mutants described earlier showed significant changes in catalytic performance under various in vitro assay conditions. Yet, in vivo performance was clearly improved. The enzymes appeared to function suboptimally in vivo due to enzyme loading with calcium, which gives poor xylose conversion kinetics. The results show that better in vivo enzyme performance is poorly reflected in kinetic parameters for xylose isomerization determined in vitro with a single type of added metal.

**Conclusion:**

This study shows that in vivo selection can identify xylose isomerase mutants with only minor changes in catalytic properties measured under standard conditions. Metal loading of xylose isomerase expressed in yeast is suboptimal and strongly influences kinetic properties. Metal uptake, distribution and binding to xylose isomerase are highly relevant for rapid xylose conversion and may be an important target for optimizing yeast xylose metabolism.

## Background

Efficient xylose conversion is an important property when selecting or engineering yeast strains to be used in second-generation bioethanol production. Fermentation of lignocellulose-derived feedstocks, which contain up to 30% d-xylose, is often carried out by *Saccharomyces cerevisiae*. Since this yeast does not metabolize the aldopentose d-xylose naturally, incorporation of either xylose isomerase or a combination of xylose reductase and xylitol dehydrogenase is necessary to convert d-xylose to the ketose d-xylulose, which can be metabolized [1–4]. The use of xylose isomerase has the advantage over the xylose reductase–xylitol dehydrogenase system that there is no intermediate production of xylitol and less formation of side products, but combining the pathways may also have certain benefits [5]. Several efforts to find suitable xylose isomerases have been reported [6–11]. A xylose isomerase discovered by Kuyper et al. [12, 13] in the fungal strain *Piromyces* E2 through genome mining (PirXI) is an attractive candidate for xylose isomerization in engineered *S. cerevisiae* strains, and is used in several studies [14]. However, in vivo performance of the enzyme is modest, as indicated by the high copy number (up to 10) of the chromosomally inserted XI-encoding gene observed in evolved strains that are capable of anaerobic d-xylose fermentation [15]. A multi-copy plasmid leading to overproduction of the PirXI protein has also been used for enhanced xylose metabolism [16]. The engineering of yeast strains showing faster xylose metabolism is an important challenge in the pursuit of strain improvement for second-generation bioethanol production [17–20].

The observation that strains with multiple copies of PirXI genes evolve during prolonged adaptation suggests that in vivo enzyme activity in *S. cerevisiae* is limiting xylose turnover [21]. Mutations in different xylose isomerases can lead to accelerated xylose metabolism and protein engineering of xylose isomerase is receiving significant attention [11, 14, 19, 20]. However, it is unclear which properties of the enzyme need to be tailored to improve its in vivo performance. A straightforward hypothesis is that the kinetic properties as reflected in catalytic rate (*k*_cat_) and/or substrate affinity (*K*_M_) at physiological conditions are not optimal for efficient xylose metabolism. On the other hand, metal affinity and in vivo metal content of xylose isomerase may also play an important role causing the enzyme to function suboptimally. Xylose isomerase is a metalloenzyme that requires two divalent metals for activity, and the wild type shows the best activity with Mn^2+^ [22]. However, metal content of the enzyme expressed in yeast may vary [21, 22]. A yeast strain with a mutation in its PMR1 gene which influences manganese homeostasis and increases Mn^2+^ content of the PirXI protein showed an enhanced rate of xylose consumption [21]. The high expression levels of the PirXI protein in selected xylose-metabolizing strains could be a burden for cell growth, and reduced expression of a more active enzyme would improve xylose consumption and growth rates. In view of the complexity of yeast cells, there may well be other factors that determine the performance of heterologously expressed enzymes, including compartmentalization, enzyme stability, and competition for metals with other cellular components.

Modern protein engineering tools enable tailoring of enzyme properties for specific applications. A particularly effective strategy is the use of directed evolution, i.e., the construction of mutant libraries and screening those for improved variants [23–25]. This approach does not require structural information. Here, one can take advantage of the fact that xylose isomerase activities limit the growth of *S. cerevisiae* on d-xylose and employ a random mutagenesis method with in vivo selection for improved growth [9, 14]. This allowed the discovery of unexpected xylose isomerase mutations, some of which were far away from the active site. It is known that distant mutations can enhance activity, e.g., by influencing enzyme surface properties [26]. On the other hand, the lack of focus in random mutagenesis protocols yields libraries with a low abundance of beneficial mutations, and a very large number of mutants often must be screened to discover better enzymes. So-called smart libraries, which incorporate phylogenetic and structural information in the design, are assumed to better cover functional sequence space, increasing the chance of discovering useful mutations and reducing the need for extensive screening [27–29].

**Fig. 1:**
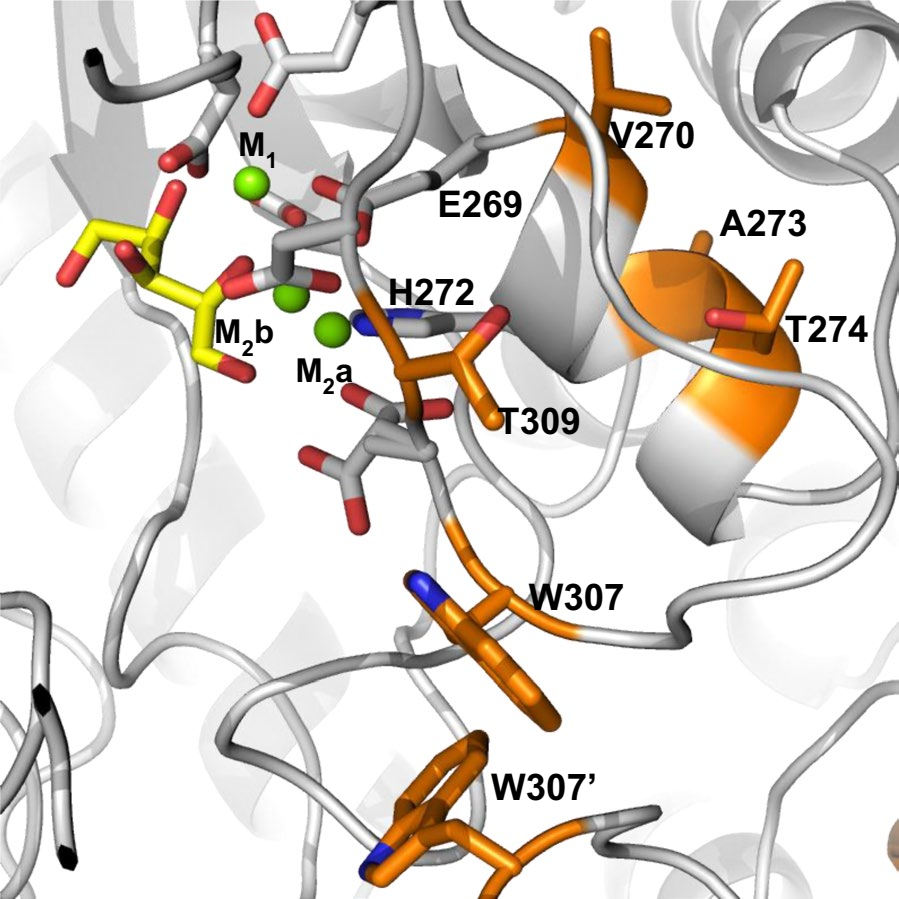
Structure of the PirXI active site and design of mutant library LibM1. The figure shows the active site structure of PirXI with xylose (yellow) and Mg^2+^ ions (green spheres) bound (PDB: 5NH7). The target residues (orange) are located near the active site. The catalytic metal (M_2_) can occupy two positions (M_2_a and M_2_b). W307′ is a residue from a neighboring subunit

To support PirXI engineering and understand the effects of selected mutations, we have recently characterized the enzyme both structurally and biochemically [22]. Even though the unidentified causes of the modest in vivo performance of PirXI and the complexity of the kinetic mechanism cause uncertainty about the types of mutation to introduce, the structures still provide useful information by revealing the residues that shape the substrate- and metal-binding sites. In the crystal structures, PirXI appeared as a homotetramer with each monomer (49.5 kDa, 437 aa) possessing an active site in which two divalent metal ions are bound. Soaking and cocrystallization studies showed that the ring-opened xylose binds in between two fully conserved tryptophan residues (Trp50 and Trp189) which play a role in the correct positioning of the substrate for catalysis [22, 30]. Of the two active site metals, one (M_1_) is responsible for substrate binding while the other (M_2_) is essential for catalysis by polarizing the M_2_-bound catalytic water that protonates O1 of the substrate and consequently generates a carbocation on C1 promoting the C2 to C1 hydride shift [22, 31]. The catalytic metal M_2_ moves during the reaction from the M_2_a to the M_2_b position, which is also visible in structures with certain combination of ligands: 5NH7 (xylose and Mg^2+^), 5NHC (xylulose and Co^2+^), 5NHD (xylose and Ni^2+^) and 5NHE (xylose and Cd^2+^) (Fig. 1) [22].

To examine if structure-inspired mutagenesis can contribute to obtaining improved xylose isomerase variants and to investigate the possibility that such mutants may be useful to identify catalytic properties relevant for in vivo performance, we now designed focused mutant libraries with mutations surrounding the residues involved in metal and substrate binding. These were screened for enhanced growth after expression in yeast. Improved enzymes were indeed discovered and their biochemical properties were investigated, in comparison to previously reported PirXI variants. The results indicate that small changes in catalytic properties may be accompanied by significant effects on xylose-supported growth. Furthermore, in vivo selection may govern mutations that improve xylose metabolism without changing kinetic properties measured under standard conditions with Mg^2+^ as the activating metal.

## Results

### Library design

To discover mutants of PirXI that enhance xylose metabolism, we designed and constructed small focused mutant libraries followed by in vivo screening for better variants. Recent structural and biochemical information was used to select target positions for mutagenesis, focusing on residues that surround the metal-binding sites. Replacing the second shell residues might have an effect on metal binding or reactivity and thereby influence the activity. The fully conserved metal-coordinating residues in PirXI are Glu233, Glu269, Asp297 and Asp340 for site M_1_ and Glu269, His272, Asp308 and Asp310 for the catalytic metal M_2_. For the first library (LibM1), we targeted residues that are in close proximity of the M_2_ site. Five residues were selected: three (Val270, Ala273 and Thr274) that lie on the same helix as the metal binding residues (His272 and Glu269), and two (Trp307 and Thr309) that are on a nearby loop (Fig. 1). Amino acid diversity to be introduced at each position was selected based on phylogenetic diversity, in silico-predicted stabilities of the mutants, and visual inspection of the predicted mutant structures. For phylogenetic input, a multiple sequence alignment was performed on 22 different class I and 100 class II XI sequences. Considering conservation scores and similarities between amino acid properties, the library diversity was decided. For example, residue Ala270 is fully conserved throughout all class II enzymes and therefore the diversity at this position was restricted to alanine and glycine to avoid extreme modifications. Changes in free energy of folding (ΔΔ*G*_fold_) of mutants relative to the wild-type enzyme were predicted using FoldX calculations [32]. Large decreases in predicted stability were used to dismiss mutations from the library design. The resulting LibM1 library included 1008 different variants (Table 1).

**Table 1:** Design of PirXI library LibM1

Residue	Amino acids	Codons	Freq. WT^a^
V270	V A G	GBT	0.33
A273	A G	GST	0.5
T274	T I K Q	AYC + MAA	0.25
W307	W F Y I S T N	WHT + TGG	0.14
T309	T S K R N Q	AVS + CAG	0.29

^a^Codons at each position were selected in such a way that each desired amino acid, including the wild type, is included at a frequency of 1/(total diversity)

### Library construction

To construct the LibM1 mutant library, at each target position a minimum number of partially undefined codons covering the selected set of amino acid substitutions was chosen using a spreadsheet implementing the CodonFinder routine [33]. The codons were selected in such a way that all the desired amino acids (including the wild-type residues) are incorporated at balanced coverage without introduction of undesired codons like stop codons. The 1008 LibM1 mutant library was covered by 8 partially undefined codons (Table 1).

Library DNA was obtained by generating gene fragments using PCR and subsequent cloning into *E. coli*–yeast shuttle expression vector (pRS426-URA) as described in “Methods”. Transformation of the library DNA to *E. coli* resulted in over 8000 clones, of which pooled plasmid DNA was transformed to *S. cerevisiae* strain DS75543, producing 6000 colonies. Considering the library size of 1008, these numbers are sufficient for near full library coverage [33]. Prior to yeast transformation, library diversity was confirmed by sequencing a mixture of plasmids isolated from the mixed collection of *E. coli* transformants. The sequencing results showed that all expected bases were incorporated at the correct positions, indicating sufficient library quality to proceed to screening.

### Screening for improved xylose utilization

Library LibM1 was screened by growth competition of *S. cerevisiae* strain DS75543 transformed with library plasmids DNA. The entire collection of yeast transformants was inoculated into xylose medium and cells were cultivated with serial transfers to fresh xylose medium. Faster growing cells, which over time became dominant, were assumed to harbor an improved PirXI. Screening was performed both under aerobic and anaerobic (oxygen-limited) conditions, each in duplicate, as different variants can be expected depending on the metabolic status of the cells. For anaerobic growth, the cultures were kept oxygen-limited as described in “Methods”. The effect of limited oxygen availability was reflected in the final cell densities (OD_600_) of the cultures, which were ~ 3 and > 20 for anaerobic and aerobic conditions, respectively. Anaerobic cultures initially required 8–9 days before growth occurred. A reduced lag time and/or increased growth rate was observed after multiple transfers with all four selection cultures, also in comparison to a control culture harboring only wild-type PirXI.

Both aerobic and anaerobic cultures were harvested after the 10th transfer and plasmids were isolated to evaluate the selected PirXI genes. Sequencing showed that all four cultures, i.e., both the aerobic duplicates and anaerobic duplicates, contained only one PirXI variant, which carried the mutations V270A and A273G.

### Effect of V270A–A273G PirXI on xylose utilization

**Fig. 2:**
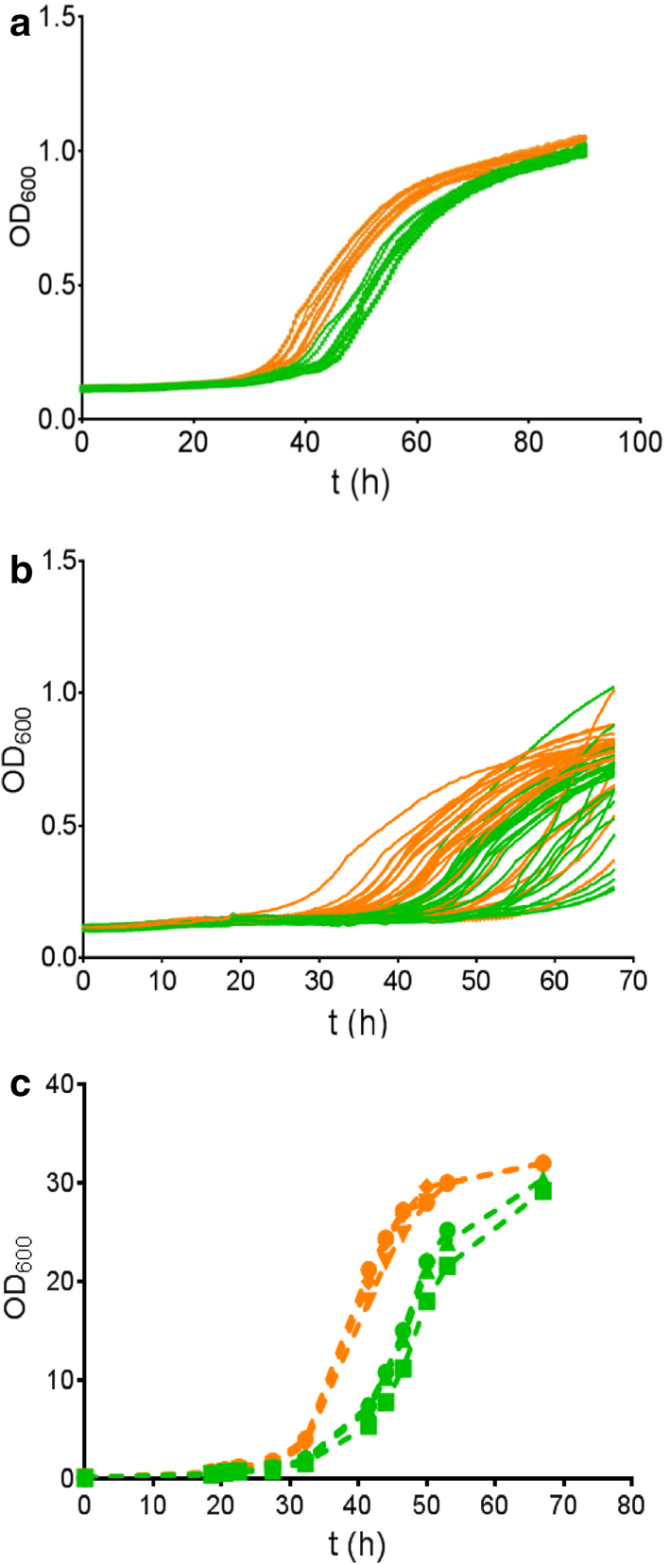
PirXI V270A–A273G improves growth of *S. cerevisiae* strain DS75543 on xylose. The orange and green lines represent growth on xylose (20 g l^−1^) of yeast expressing the mutant and the wild-type PirXI, respectively. **a**, **b** Growth in 96-well plate with continuous measurement of optical density. Each line represents an individual clone selected from a transformation plate. **c** Growth in triplicate in batch culture with intermittent sampling and measurement of optical densities

The consistent selection of the V270A–A273G variant from library LibM1 suggested that the screening method was reliable and sensitive. Nevertheless, it is possible that other events such as genomic mutations or variations in expression level were responsible for the improved growth of yeast carrying the V270A–A273G mutant PirXI. The replicon of the pRS416 vector used in this work is derived from yeast plasmid 2*µ*, and plasmid copy numbers can vary from culture to culture [34]. Furthermore, in laboratory evolution of *S. cerevisiae* for growth on xylose, cells may acquire diverse chromosomal mutations that cause improved growth [35, 36]. To prove that in our case the selected mutations in the PirXI structural gene caused improved growth on xylose, the mutations were reconstructed by site-directed mutagenesis in the original PirXI gene and cloned in a vector that was not subjected to previous selection. *S. cerevisiae* DS75543 cells were then transformed with the freshly prepared constructs and their growth performance in a 96-well plate was monitored. We have repeated this process several times and consistently observed that the cells containing the mutant PirXI grow on xylose better compared to those containing wild-type PirXI (Fig. 2a). We have also observed that general growth performance of the cells slightly differs between experiments as well as between clones picked from a single transformation experiment. Figure 2b shows a high variability in growth between 30 independent transformants despite their identical genotype. Nevertheless, the results clearly indicate that the V270A–A273G mutant PirXI improves growth on xylose as compared to wild type, especially in the earlier phases of growth (Fig. 2b). On average, the mutant cultures started to grow earlier and more quickly reached their final density.

In 96-well plates, oxygen availability may not be well controlled and results could be influenced by evaporation. Therefore, a comparison between the wild-type and V270A–A273G XI variants was also performed using shake flask cultures with replicates inoculated with pre-cultures from independent transformants. The resulting growth curves (Fig. 2c) confirm that the mutated PirXI is beneficial for growth on xylose. The specific growth rates (*µ*) were calculated from the exponential part of the curves, using the following equation for fitting: ln*X* = ln*X*_*0*_ + *µ*(*t* − *t*_0_), where *X* is the measured OD_600_ and *µ* is the rate. The average growth rates of the wild type and the mutant are 0.13 ± 0.01 and 0.18 ± 0.01 h^−1^, respectively. These results show that the observed improved growth is due to the V270A–A273G mutations in PirXI, not by unidentified mutations elsewhere on the plasmid or in the chromosome of the selected transformants.

**Fig. 3:**
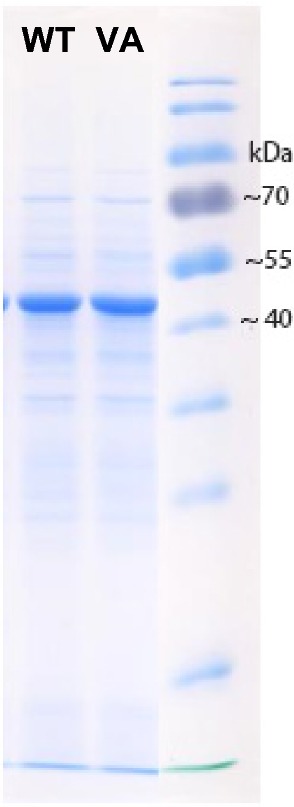
Expression level and cell-free extract activities of PirXI variants. Samples of 2 µg of crude extract protein prepared from cells grown on xylose medium were loaded on an SDS gel. The dominant bands at ~ 50 kDa represent PirXI. The expression level calculated by measuring the intensity of the bands is about 25% for both samples. WT: wild-type PirXI VA: V270A–A273G PirXI

Yeast cells selected for good growth on xylose show high overexpression of PirXI [12, 15], which may be a metabolic burden for the cells and trigger selection of mutants with a higher activity:expression ratio in competition experiments. To examine if the PirXI mutations affected enzyme expression, we studied XI levels in cells grown on xylose. The specific activity with 100 mM xylose measured with the cell-free extracts were 0.94 U/mg and 0.54 U/mg for the wild type and the mutant, respectively. SDS-PAGE gels revealed that the expression levels for the wild-type and the mutant enzyme were similar (Fig. 3).

The uncertainty of in vivo metal binding properties of PirXI and metal content of the yeast cytoplasm makes it difficult to define a metal composition for assays that gives results reporting on in vivo performance. When we measured the PirXI activity of extracts of *S. cerevisiae* DS75543 cells without metal addition, the results indicated that the activity of the wild-type enzyme was almost twofold higher compared to the mutant. This unexpected observation could be caused by changes in PirXI metal composition during enzyme preparation and dilution, e.g., due to binding of metals released from organelles such as vacuoles or from changes in metal–protein interactions.

**Fig. 4:**
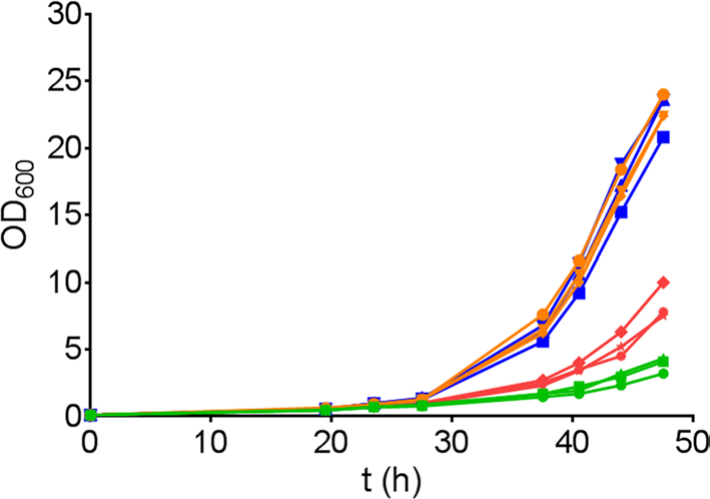
Effect of the mutations V270A and A273G on growth. Growth in xylose medium of *S. cerevisiae* cells containing PirXI variants was followed. Wild type (green lines), V270A (blue lines), A273G (red lines) or V270A–A273G (orange lines). Measurements were performed in triplicate, shown as individual lines

To examine if the individual mutations in the PirXI variant are both necessary for improved growth, we constructed the single mutants V270A and A273G and examined the effect on growth on xylose, particularly on the early growth phase. The growth curves indicate that the V270A mutation has a larger effect, showing much earlier initiation of exponential growth (Fig. 4), but also cells containing the A273G mutant PirXI showed a slight growth improvement compared to the cells expressing the wild-type enzyme.

### Kinetic properties of PirXI V270A–A273G

We expected that the positive effect of the V270A–A273G mutations on xylose-supported growth would be due to improved catalytic parameters, i.e., increased catalytic rate (*k*_cat_) or a better substrate affinity (reduced *K*_M_). Since PirXI can be activated by different divalent metals and activities depend on the type of metal that is bound [22], we measured XI activities with metals that the enzyme potentially binds in vivo as previously found by metal analysis (Mg^2+^, Mn^2+^ or Ca^2+^) [21]. With none of these metals, the in vitro activities revealed an increased *k*_cat_ or decreased *K*_M_ for the mutant enzyme in comparison to wild type. In contrast, the wild type performed better in the presence of all metals tested, showing slightly higher catalytic rates and substrate affinities (Table 2). Especially, with Mn^2+^, the activity of the mutant decreased 50% compared to the wild type. This result indicates that an increase in specific activity with these metals, at least individually, is not responsible for the improved growth on xylose of yeast expressing V270A–A273G PirXI.

**Table 2:** Kinetic parameters of PirXI variants

PirXI variants	Metal	*K*_act_ (µM)^c^	Kinetic parameters^a^		
			*k*_cat_ (s^−1^)	*K*_M_ (mM)	*k*_cat_/*K*_M_ (s^−1^ M^−1^)
WT	Mg^2+^	130 ± 7	2.2 ± 0.1	7.1 ± 0.5	310
	Mn^2+^	0.44 ± 0.05	6.9 ± 0.2	6.2 ± 0.7	1110
	Ca^2+^	230 ± 20	0.8 ± 0.1	1360 ± 160	0.4^b^
V270A–A273G	Mg^2+^	160 ± 14	1.8 ± 0.1	11.6 ± 1.6	155
	Mn^2+^	0.39 ± 0.05	2.8 ± 0.1	9 ± 1	310
	Ca^2+^	140 ± 12	0.28 ± 0.01	1760 ± 120	0.18^b^
S141N–T142S–A143S–G174A	Mg^2+^	126 ± 10	1.6 ± 0.2	27 ± 9	60
	Mn^2+^	2.4 ± 0.5	4.9 ± 1	37 ± 1.5	132
	Ca^2+^	96.5 ± 3.5	0.06 ± 0.01	1200 ± 300	0.03^b^
E15D–T142S	Mg^2+^	190 ± 10	1.9 ± 0.1	22 ± 2	86
	Mn^2+^	2.1 ± 0.3	5 ± 0.2	53 ± 4	94
	Ca^2+^	50 ± 10	> 0.02	N.A.	0.018^b^
N338C	Mg^2+^	112 ± 7	4.2 ± 0.2	22 ± 2	191
	Mn^2+^	1.3 ± 0.3	7.2 ± 0.2	29 ± 1.5	248
	Ca^2+^	163 ± 6.5	0.78 ± 0.2	2800 ± 800	0.2^b^

^a^All assays were performed at 30 °C in 20 mM MOPS buffer, pH 7. Values are averages from three (wild type, V270A–A273G PirXI) or two (S141N–T142S–A143S–G174A, E15D–T142S and N338C PirXI) independent measurements. The margins represent standard deviations

^b^Catalytic efficiencies obtained from slopes of Michaelis–Menten plots at [*S*] ≪ *K*_M_ because of the high *K*_M_ for xylose in the presence of Ca^2+^ as activating metal

^c^The *K*_act_ value is defined as the metal concentration giving half-maximal activity, as measured with a saturating concentration of xylose (100 mM in case of Mg^+^ and Mn^2+^, 400 mM in case of Ca^2+^)

Besides the metal-dependence of the isomerase, metal affinities were considered as a possible cause of improved in vivo enzyme performance. We estimated metal affinities of the wild-type and the mutant enzyme by measuring the activation constant (*K*_act_) for each metal. This constant represents the metal concentration giving half-maximal enzyme activity. Since xylose isomerase requires two metals for activity, the value depends on the binding sites with the lowest affinity if both sites must be occupied. For measuring *K*_act_ with Mg^2+^ and Mn^2+^, 100 mM xylose was used as substrate. In case of Ca^2+^, 400 mM xylose was used since the *K*_M,xylose_ of the PirXI-Ca^2+^ is very high (Table 2). The data showed that the V270A–A273G mutant showed slightly higher affinity for Ca^2+^ and Mn^2+^, whereas the wild-type enzyme has slightly higher affinity for Mg^2+^ (Table 2). However, the differences are small and do not indicate a shift in metal affinity as the cause of improved growth.

**Fig. 5:**
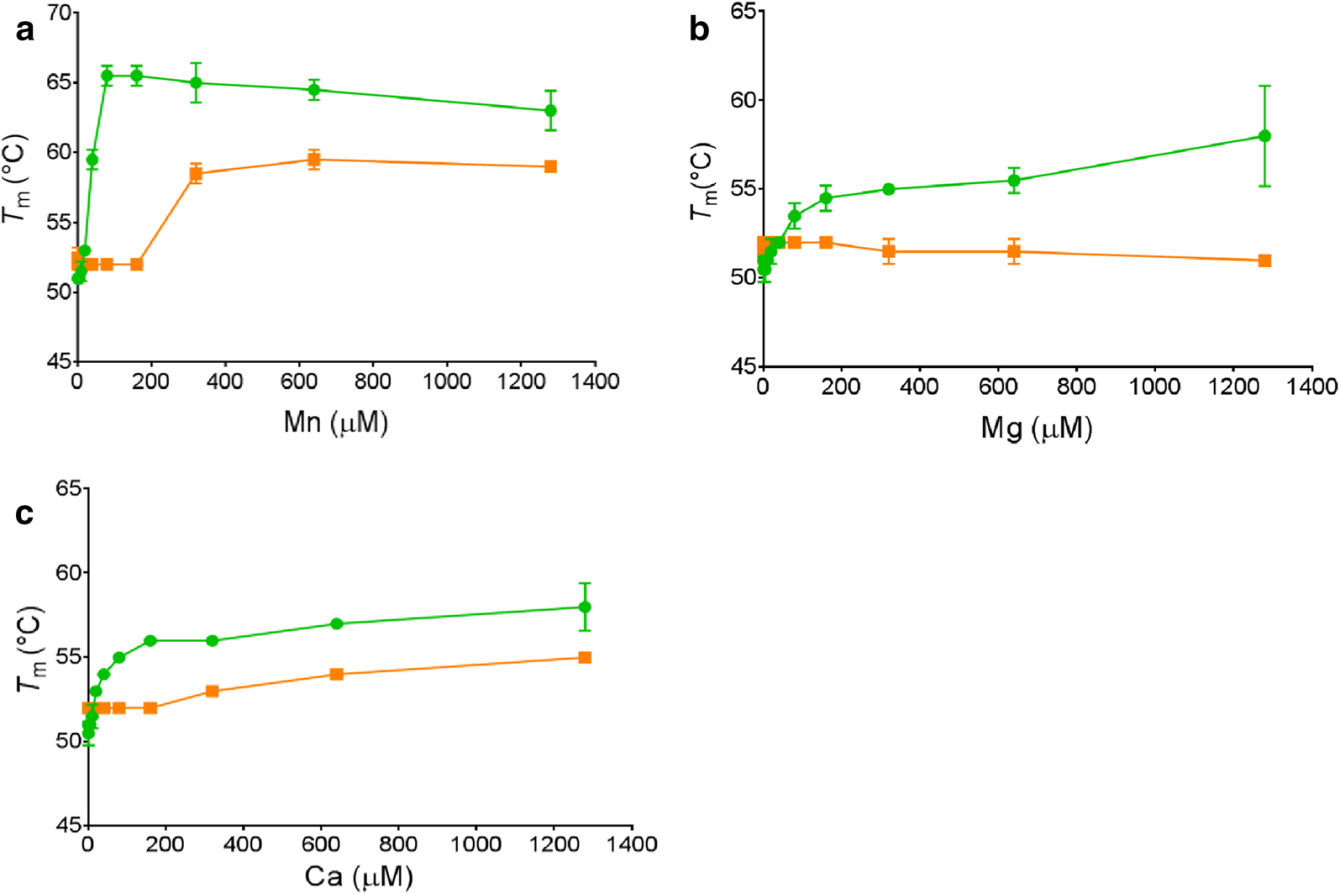
Effect of metals on thermostability of PirXI. The graphs show the apparent melting temperatures of purified and EDTA-treated wild-type PirXI (green lines) and the V270A–A273G mutant PirXI (orange lines) in the presence of various concentrations of metals (**a** Mn^2+^; **b** Mg^2+^; and **c** Ca^2+^). The *T*_m_ values are averages from two independent measurements. [*E*] = 20 μM. Error bars represent standard deviations

Metal affinity was also examined by measuring the effect of metal addition on PirXI thermostability since metal binding can stabilize metalloenzymes [37]. Effects on apparent melting temperatures were measured in the presence of different concentrations of metals using thermal shift assays (Fig. 5). The results showed that the apo forms of wild-type and V270A–A273G PirXI have a similar thermostability. Interestingly, whereas *T*_m,app_ of the wild-type PirXI increased with metal concentration according to a hyperbolic saturation-like curve, the *T*_m,app_ of the mutant enzyme was constant up to ca. 200 µM of Mn^2+^ or Ca^2+^ added, with an increase at higher metal concentrations (Fig. 5a, c). In contrast, when Mg^2+^ was added, the thermostability of the mutant enzyme was not increased even at concentrations that were saturating for enzyme activity (Fig. 5b). The difference between the metal-concentration dependence of mid points of thermal shift assays and *K*_act_ values measured in the presence of substrate suggests that substrate influences metal binding, as also observed when examining X-ray structures of the enzyme with different combinations of ligands [22]. Only in the presence of the substrate xylose both metal-binding sites in the crystal structures were occupied, whereas in xylitol- or glycerol-bound enzyme only the M_1_ metal site was occupied with a metal ion.

The metal content of yeast cells is complex and consists of both free metal ions and metal ions bound to macromolecules [38]. In general, in vivo metal binding by metalloproteins is controlled by mechanisms such as intracellular metal homeostasis, localization of protein folding, and activities of metal transporters and metallochaperones [39]. In a previous study, we showed that changes in intracellular metal composition affect metal composition of PirXI, which in turn influences catalytic performance [21]. PirXI isolated from yeast grown on xylose is mostly bound with Ca^2+^, which barely activates the enzyme. Therefore, a large portion of PirXI does not contribute to in vivo conversion of xylose. In contrast, the smaller fraction of PirXI that is bound with the strongly activating Mn^2+^ contributes most to the in vivo enzyme activity [21].

**Fig. 6:**
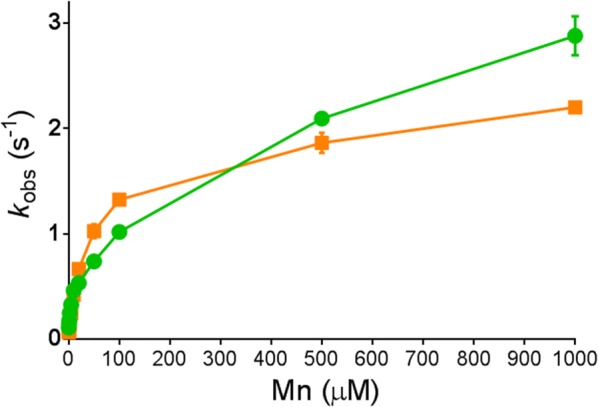
PirXI activity in the presence of Ca^2+^ and Mn^2+^. The activities of wild-type enzyme (green line) and the variant V270A–A273G (orange line) on 100 mM xylose were measured in the presence of a mixture of 1 mM Ca^2+^ and various concentrations of Mn^2+^. The data represent the average values from duplicate measurements and the error bars represent standard deviations

In view of the complex metal composition of *S. cerevisiae*, we measured the activities of the wild type and the V270A–A273G mutant in the presence of varying concentrations of Mn^2+^ and a fixed high concentration (1 mM) of Ca^2+^ (Fig. 6). As expected, both variants showed higher activity with increasing concentration of Mn^2+^. Interestingly, the degree to which Mn^2+^ influenced the activity was different between the wild-type and the mutant enzyme. At low concentrations of Mn^2+^ (10–100 µM) and in the presence of 1 mM Ca^2+^ the mutant enzyme showed slightly higher activity. This indicates that the activation of the mutant enzyme by Mn^2+^ in the presence of a high concentration of Ca^2+^ is improved. Even though the activity of the mutant is lower in the presence of Mn^2+^ or Ca^2+^ alone, at certain low Mn^2+^/Ca^2+^ concentration ratios, the V270A–A273G mutant enzyme is better activated than the wild type. These results suggest that differences in in vivo metal activation may be responsible for the improved growth of yeast cells expressing the V270A–A273G mutant PirXI.

### Crystal structures of PirXI wild type and V270A–A273G

**Fig. 7:**
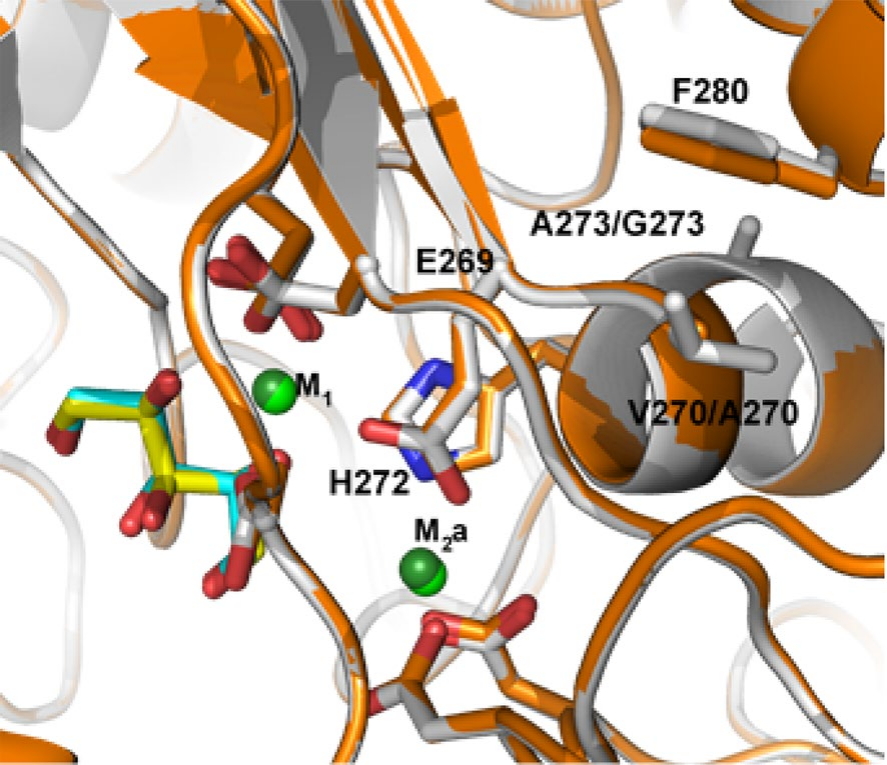
Structural alignment of active sites of wild-type and V270A–A273G PirXI. The X-ray structures are of wild-type (gray) and V270A–A273G mutant (orange) PirXI, both isolated from yeast cells grown on xylose. Ligand colors: xylose (yellow—wild type, cyan—mutant); metals (light green—wild type, dark green—mutant)

To examine possible structural changes in the V270A–A273G PirXI, we solved and compared crystal structures of this variant and the wild-type enzyme purified from yeast (Fig. 7). The overall structures of the wild type and mutant enzyme are very similar and confirmed the mutations. In the mutant structure, the side chain of Phe280 moves ~ 0.5 Å in the direction of Ala270. Due to the decrease in hydrophobicity and size the surrounding waters also shift towards Ala270. Mutation A273G shows no effect on the structure. There is no significant difference between the two structures to explain the improved in vivo performance of the mutant.

The enzyme crystals were prepared without removal or addition of metal ions so that only intrinsic metal ions are present. When the metals ions were refined as Mg^2+^, the Fo–Fc map showed unaccounted electron density at the metal positions, suggesting the presence of heavier ions. In an anomalous electron density map a clear signal was observed at the M_1_ and M_2_a positions with *σ* levels of 4.8 and 3.5, respectively. Mg^2+^ ions do not have an anomalous signal at the in-house used wavelength of 1.54 Å. However, a comparison with anomalous maps of previously determined structures [22] shows similar peak heights in the Ca–xylose structure of PirXI (PDB code 5NH8). Therefore, metal ions at the M_1_ and M_2_a positions were refined as Ca^2+^ with 100% occupancy resulting in a flat Fo–Fc map in both the wild-type and the double-mutant structures. The temperature factors (B-factors) of the two Ca^2+^ ions are 11.9 and 16.4 for the wild type and 11.4 and 19.3 for the mutant, which are lower than those of the surrounding residues. The distances of the coordinating side chains to the M_1_ ion in the wild type PirXI and the mutant enzyme isolated from yeast are similar to those in the wild-type Ca–xylose structure reported earlier (5NH8). These results indicate that most of the metal-binding sites of PirXI isolated from yeast are occupied by poorly activating Ca^2+^ ions, both in the wild-type and in the PirXI V270A–A273G mutant enzyme. Other metal ions, such as Mg ^2+^, Fe^2+^, Mn^2+^ or Co^2+^, may be bound with low occupancy.

### Construction and screening of library LibM2

A second library design for discovery of better xylose isomerase mutants focused on mutations in a stretch of six residues flanking the substrate binding site. In this case, to avoid the risk of improved growth by chromosomal mutations, we compared growth properties of yeast clones transformed with the library DNA (Table 3). The growth of library colonies on solid medium containing xylose as sole carbon source was monitored by visual inspection. Plasmid DNA was isolated from suspected positive (larger) clones, retransformed to yeast and rescreened.

**Table 3:** Library LibM2 variants and codons

Residue	Variants	Codons	Freq. WT
S141	S I V A T G N	RBC + AAC	0.14
T142	T S	ASC	0.5
A143	A T G S	RSC	0.25
N144	N L Q T	CWG + AMC	0.25
V145	V L I C	VTT + TGC	0.25
G147	G A T S	RSC	0.25

**Fig. 8:**
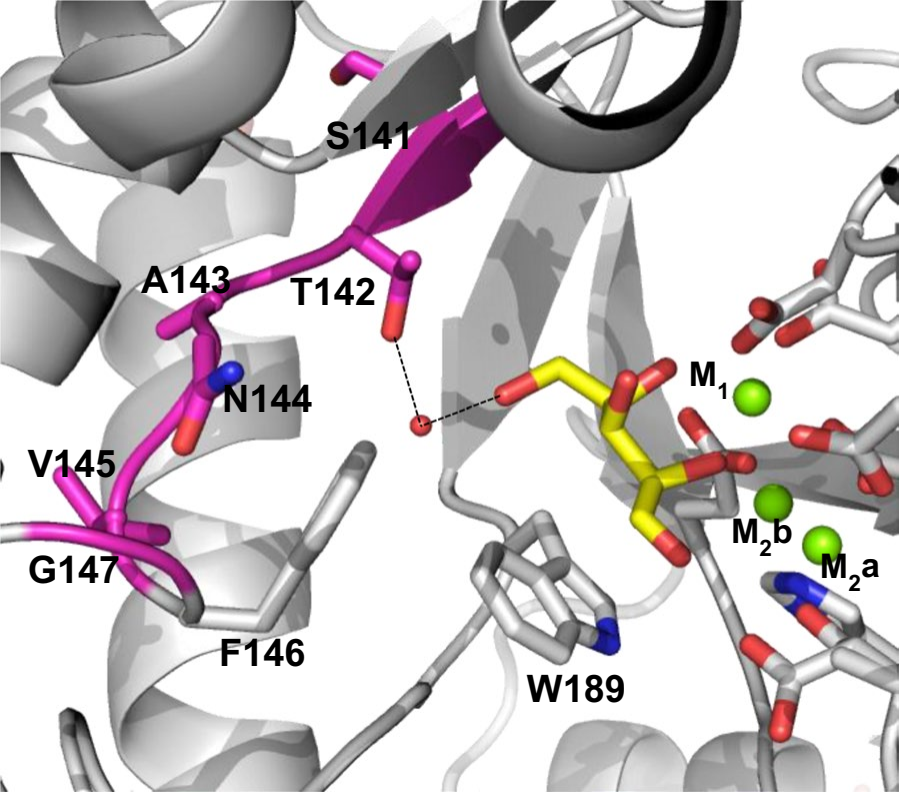
Target residues for library LibM2. Residues mutated in library M2 (magenta) surround the substrate binding site. Residue Thr142 and xylose (yellow) may interact (dashed lines) via a water molecule (red sphere). Magnesium ions and the coordinating residues are depicted as green spheres and gray. The metal binding residues and conserved active site hydrophobic residues Phe146 and Trp189 are shown as gray sticks. PDB 5NH7

Library design again included selection of target positions and diversity to be introduced at each position (Fig. 8). The residues at the six target positions (Ser141, Thr142, Ala143, Asn144, Val145 and Gly147) at the C5 side of the substrate interact with the substrate either directly or indirectly. Therefore, it was expected that modifying these residues can improve substrate binding and the catalytic rate. Residue Thr142 is fully conserved throughout all known xylose isomerase sequences. In the structure it is connected to O5 of the substrate via a water molecule. To keep this interaction, we limited the diversity at this position to Thr and Ser. In a previous study, the PirXI T142S mutation was discovered to improve the growth of yeast on xylose [14]. We preserved Phe146 as it is fully conserved and it plays an important role in keeping the active site hydrophobic. Together with Trp189 and other hydrophobic aromatic residues (Trp50 and Phe61) this promotes the hydride shift by shielding the hydride from solvent [40–42]. The resulting library consists of 3584 variants (Table 3).

**Fig. 9:**
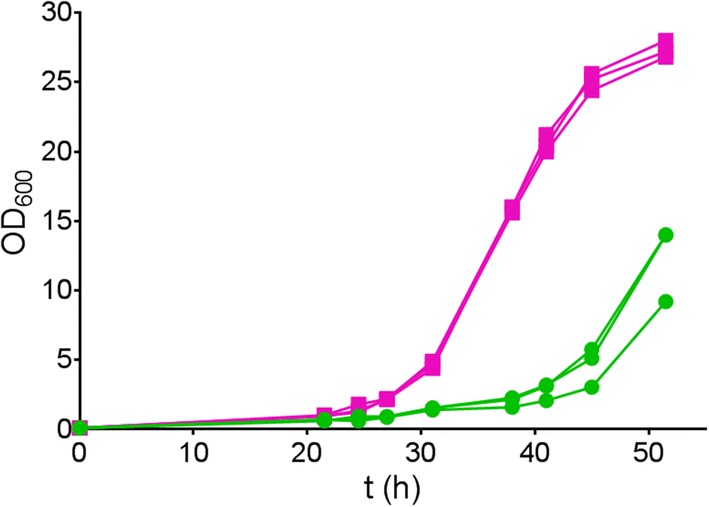
PirXI mutant S1 (S141N–T142S–A143S–G147A) improves growth on xylose. Growth of *S. cerevisiae* DS75543 cells harboring wild-type PirXI (green) or mutant PirXI S1 (magenta) on xylose (20 g l^−1^). Each line represents a biological replicate. The calculated average growth rates were 0.13 ± 0.01 h^−1^ and 0.18 ± 0.01 h^−1^ for the wild type and the mutant, respectively

The library was constructed using the same strategy as for library LibM1. The initial *E. coli* transformation yielded ca. 8000 colonies and the diversity was confirmed by sequencing a plasmid mixture obtained from pooled transformants. The subsequent transformation to *S. cerevisiae* DS75543 also resulted in over 8000 colonies. For identification of clones showing improved xylose utilization, transformed cells were washed from glucose plates and spread on xylose plates (see “Methods” for details). Many cells did not grow at all or started to grow very slow, causing visible differences between individual colonies, also for wild type. The latter indicated that other factors than PirXI activity influenced colony growth, for example the physiological status of transformed cells at the moment of plating. After three rounds of screening and retransformation, 46 colonies which showed superior growth were selected. To identify the best variant, growth in xylose containing liquid medium was measured using 96-well plates and compared to wild type. Most of the 46 variants reproducibly showed improved growth. The xylose isomerase genes from the 24 best growing variants were sequenced, revealing 10 different variants, one of which was wild type. The sequences that appeared most frequent (4–8 times) were reconstructed in a clean background and the effects on growth on xylose were evaluated after transformation to fresh DS75543 cells. Among these mutants, variant S1 (S141N–T142S–A143S–G147A) consistently showed the biggest improvement of growth on xylose when several independent cultures were tested. As shown with variant V270A–A273G, the most significant effect of mutant S1 also appears to be on the earlier start of the growth while showing a slightly increased exponential growth rate (Fig. 9).

### Kinetic properties of PirXI S1

The reconstructed PirXI mutant S1 was purified from *E. coli* and its activity was measured after reconstitution with different metals. As with variant V270A–A273G, the Michaelis–Menten kinetic parameters measured in the presence of Mg^2+^, Mn^2+^ or Ca^2+^ revealed reduced *k*_cat_ values as compared to wild type (Table 2). The *K*_M_ for xylose was also several fold higher in the presence of Mg^2+^ or Mn^2+^ compared to the wild type. Furthermore, the *K*_act_ values indicate that a shift in metal affinity does not promote better in vivo performance of the mutant enzyme as the affinity towards the most activating metal Mn^2+^ decreased, while the affinity towards Ca^2+^, which poorly activates the enzyme, increased.

### Performance of other xylose isomerase mutants

The results described above indicate that both libraries yielded PirXI mutants that caused accelerated growth on xylose. However, their properties exhibited disconnection between in vivo performance and in vitro catalytic properties. We further explored the ambiguous relation between in vivo and in vitro enzyme properties by studying PirXI mutants discovered independently in previous studies, using different *S. cerevisiae* host strains [9, 14]. Using directed evolution with random mutant libraries, Lee et al. discovered PirXI variant E15D–T142S which increased the growth rate on xylose from 0.01 h^−1^ up to 0.06 h^−1^ [14]. Activities of these enzymes have only been measured with cell lysates, making a comparison difficult, but *K*_M_ values appear high. Later, Katahira et al. discovered that mutations at position N338, especially substitution N338C, improved growth of yeast on xylose. This mutation was effective not only in PirXI but also in related XIs [9]. It was reported that yeast cells carrying the N338C variant of PirXI consumed xylose 3–4 times faster than cells carrying wild-type PirXI. The catalytic properties of the mutant enzymes from these studies have not been described. Very recently, when our study was nearing completion, Seike et al. described mutations in XI from *Lachnoclostridium phytofermentans* (LpXI) that enhanced d-xylose metabolism [11]. The most effective mutations were T63I and V162A. The corresponding positions in PirXI are distant from the active site and the mutations were not examined here.

**Fig. 10:**
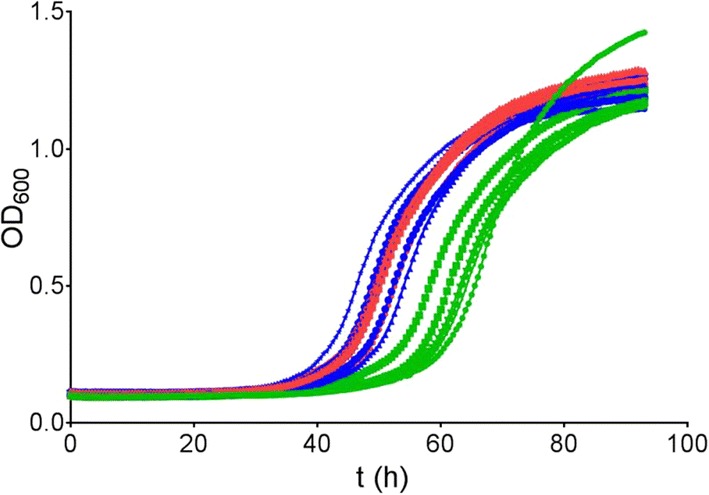
Growth of *S. cerevisiae* expressing PirXI variants E15D–T142S or N338C. Growth of cells expressing the wild-type PirXI (green lines), a mutant PirXI E15D–T142S (red lines) or N338C (blue lines) on xylose (20 g l^−1^) medium were measured using a microtiter plate reader. Individual lines represent biological replicates

We constructed the two earlier PirXI mutants [9, 14], E15D–T142S and N338C, and confirmed that the variants are beneficial for PirXI-mediated growth of strain DS75543 on d-xylose as well (Fig. 10). Subsequently, we expressed the mutants in *E. coli*, purified the enzymes and measured kinetic parameters. For this, activities were determined with Mg^2+^, Mn^2+^ or Ca^2+^ added to the apoenzyme and the activation constants were also determined (Table 2). As with the new mutants described in the current paper, Michaelis–Menten parameters and metal affinities did not reflect the positive effect of the mutations on growth, with the exception of an increased *k*_cat_ of the N338C mutant in the presence of Mg^2+^ and Mn^2+^. However, this enzyme also has a higher *K*_M_. This result shows that the disconnection between in vivo performance and in vitro properties of PirXI is not dependent on the screening strain or selection conditions.

## Discussion

Improving d-xylose metabolism is an ongoing challenge for optimizing second-generation bioethanol production by engineered strains of *S. cerevisiae*. Important improvements in yeast performance have been achieved by evolutionary and metabolic engineering, which can influence different steps in central metabolism [16, 43], and by enhancing the xylose uptake system [44, 45]. Introduction of efficient enzymes for initial isomerization of d-xylose to d-xylulose is an equally important target. The incorporation of *Piromyces* xylose isomerase in xylulose fermenting yeast strains allowed xylose utilization [12, 13], but very high expression levels are needed for optimal performance, as indicated by gene amplification up to over 10 copies during adaptation [15, 21], leading to production of xylose isomerase at up to 25% of the cellular protein (Fig. 3). This suggested that the enzyme has poor in vivo kinetics and stimulated research aimed at discovering better xylose isomerase variants [9, 14].

In view of the catalytic properties of PirXI, the need for such a high expression level is unexpected. At the observed xylose isomerase content of 25%, one would expect the isomerization reaction not to be growth-limiting. The relation between growth rate *μ* and xylose consumption *V* can be expressed as:

$$ \mu = V \cdot Y ,{\text{ with}} $$

$$ V = [E] \cdot \frac{{V_{ \hbox{max} } \cdot \left[ S \right]}}{{K_{\text{M}} + \left[ S \right]}} , $$

where [*E*] is the enzyme content (ca. 0.1 g PirXI per g biomass estimated based on on 25% of the total protein being XI, and a total protein content of 0.4 g per g biomass [46, 47]), *V*_max_ (8.3 U/mg, from *k*_cat_ = 6.9 s^−1^ with Mn^2+^) and *K*_M_ (6.2 mM) have their usual meaning. *Y* represents the yield on xylose (0.25 g cell dry weight/g xylose converted) estimated from a previous study performed with *S. cerevisiae* grown in a similar condition [48]. This predicts a PirXI activity at [*S*] = 1–10 mM (reasonable intracellular substrate concentration) [49, 50] of 1–4.6 g xylose converted per g biomass per h, allowing a growth rate of *μ* = 0.25–1.15 h^−1^. The experimentally observed growth was around 0.13 h^−1^, suggesting that xylose isomerase should not be rate limiting if it were fully active. PirXI is also well expressed and folded in vivo, which may be troublesome with other XIs, as illustrated by the extreme case of the xylose isomerase from *Actinoplanes missouriensis*, which in vitro looks catalytically superior to PirXI but fails to function in *S. cerevisiae* [6, 51]. In view of earlier work on the effect of metals on PirXI activity and the observation that mutations influencing manganese homeostasis can improve xylose metabolism, we initially suspected that the modest activity of the enzyme might be due to suboptimal metal loading and that xylose utilization could be improved by engineering PirXI variants carrying mutations surrounding the metal-binding sites.

The discovery and engineering of XI variants that improve xylose metabolism has been pursued by different groups [9, 11, 52]. However, the connection between in vitro kinetics of xylose isomerase and yeast growth on xylose remains rather unclear. Recently, Seike et al. [11] compared xylose isomerases from different organisms and found that an enzyme from *L. phytofermentans* (*Lp*XI) and two mutants thereof gave the highest xylose consumption rate even though its activity measured in cell lysates were not better than those of cells expressing PirXI [7]. Mutants of *Lp*XI that gave better xylose consumption were found. A higher activity (*V*_max_) and lower *K*_m_ were found with a double mutant of *Lp*XI, but the experiments were done with whole cell lysates reconstituted with Mg^2+^, so a comparison of intrinsic kinetic parameters is difficult. Similarly, an XI from *Burkholderia cenocepacia* which gave higher in vitro activity of cell lysates compared to PirXI and LpXI [52] did not seem beneficial for xylose fermentation [11]. In the same context, a recently discovered XI from the gut bacterium *R. speratus* was found to be better for xylose fermentation than PirXI, but this could not be explained by differences in in vitro catalytic performance [9].

We initially expected that selection of faster growing yeast strains from libraries expressing mutants of PirXI would give variants with improved kinetic parameters (higher *k*_cat_, lower *K*_M_) with Mn^2+^. Also changes in metal binding affinity or shifts in metal preference could be expected. Two focused libraries with good diversity at the target positions were constructed and improved mutants were indeed obtained by batch culture selection and plate screening for higher growth rates. From the first library (LibM1), the same V270A–A273G PirXI variant was repeatedly retrieved, both from aerobic and anaerobic duplicate cultures. This indicates that the features of PirXI which limit growth of yeast on xylose are not dependent on oxygen availability, and that aerobic screening is possible to discover mutations that contribute to anaerobic xylose metabolism as well. Examining a reconstructed V270A–A273G PirXI mutant demonstrated that the improved performance was due to the mutations in the PirXI structural gene. A second focused library (LibM2) was screened on solid xylose medium and led to the discovery of the fourfold mutant S141N–T142S–A143S–G174A. Again, the contribution of chromosomal mutations was excluded. After discovery of these new mutants, we investigated the relation between enzyme kinetics and improved growth, an issue that is also still open for earlier mutants of PirXI which were obtained by enrichment after error-prone PCR [9, 14]. With the reconstructed mutant genes expressed in a clean expression vector and host, we found that all four PirXI variants improved growth on xylose of *S. cerevisiae* DS75543, a strain different from the one used earlier by others [9, 14].

Using purified proteins of the two new mutants (V270A–A273G and S141N–142S–A143S–G174A) as well as of the earlier variants (E15D–T142S and N338C) [9, 14], we measured activities and kinetic parameters under a variety of conditions. Activities at physiological pH (~ 7) and temperature (30 °C) initially did not reveal any obvious features of the mutant enzymes that account for faster growth on xylose. Measurements with enzyme variants that were reconstituted with a single type of metal indicated that the mutants under these conditions had no advantage over the wild type, but likely this incompletely reflects in vivo conditions where the enzyme binds mixtures of metals and needs to function in the complex environment of the cytoplasm. Metal binding in the cytosol of *S. cerevisiae* is dependent on metal availability as well as binding affinities, which differ among xylose isomerases and between the two binding sites [53, 54]. The most pronounced yet small effect was the increased *k*_cat_ of the N338C mutant with Mn^2+^ and Mg^2+^ as activating metals, as well as a slightly improved activity of the V270A–A273G variant in the presence of a low concentration of Mn^2+^ and a high concentration of Ca^2+^. Thus, at certain concentrations of these two metals, the mutant enzyme can be more active than the wild type. With all PirXI variants examined, Mn^2+^ gave much better activity than Ca^2+^, emphasizing the importance of Mn^2+^ homeostasis for enzyme activity, which was also shown in our previous study where cellular manganese content was enhanced by mutations in a metal transporter [21].

We also found that the main metal in PirXI isolated from yeast is Ca^2+^ and only a small amount of Mn^2+^ is present [21]. This metal composition of the enzyme is far from optimal for catalysis as Ca^2+^-bound PirXI shows very high *K*_M_ for xylose, over 200-fold higher than with the catalytically preferred Mn^2+^ (Table 2). A shift in metal preference thus can explain improved in vivo performance, although establishing a quantitative correlation is impossible since in vivo metal binding of the enzyme is difficult to predict and measure, especially because the enzyme has two metal-binding sites with different affinities, with one site essential for catalysis yet probably only occupied when substrate is bound [22]. Indeed, the fact that the metal composition of PirXI does not strictly follow the apparent metal affinities (*K*_act_) and intracellular metal composition of *S. cerevisiae* indicates a complex in vivo metal binding mechanism of the enzyme [21]. Changes in metal binding were also suggested by a different response of the wild-type and mutant PirXIs to metal titration followed by thermal shift assays. While the thermostability of wild type increases with increasing metal concentrations, the mutant V270A–A273G required high concentrations of the metals (> 200 µM) for an effect on thermostability, illustrating that the typical increase in thermostability of a metalloenzyme upon metal binding is abolished by the mutations. It is possible that binding of metals is affected by the availability and binding of substrate [22, 40].

Even though the mutations changed the metal specificity of the enzyme, increasing the affinity for both Mn^2+^ and Ca^2+^ slightly, crystal structures of mutated PirXI isolated from yeast showed that the enzyme still has Ca^2+^ ions occupying both metal-binding sites. It is interesting that the structure of PirXI isolated from *E. coli* showed a metal composition quite different from that of the enzyme produced in yeast, with in case of the yeast enzyme only the M_1_ binding site being occupied by Fe^2+^ (25%), Ca^2+^ (40%) and Mg^2+^ (35%) as estimated from structural refinement and with the M_2_ site left empty. These data confirm that in yeast most of the enzyme is loaded with the catalytically impractical Ca^2+^ ions, with a high impact on the in vivo performance of the enzyme. This indicates that conclusions about increased *V*_max_ values of evolved XIs should be considered with care, especially in case of measurements performed with cell-free extracts instead of purified enzyme and in the presence of an excess of added Mg^2+^, which is routinely used in XI assays [11, 14].

Other factors that might possibly influence in vivo enzyme performance include enzyme stability (lifetime), compartmentalization, and interaction with other cellular macromolecules. PirXI originates from a heterologous host causing the enzyme to be not evolutionarily optimized for functioning in *S. cerevisiae*. The distribution of metals over cellular compartments and the sequestration of metals by other macromolecules are likely to have a major impact on the metal availability for PirXI in vivo [55]. Such differences in enzyme properties beyond kinetic parameters may influence the performance of xylose isomerase variants. Differences between host strains, as well as variations in cultivation conditions and assay conditions make it difficult to compare the performance of xylose isomerases in yeast xylose metabolism.

## Conclusion

As part of developing efficient second-generation bioethanol production, there have been efforts to engineer xylose isomerase variants that improve growth of *S. cerevisiae* on xylose. We found that design of focused libraries of *Piromyces* xylose isomerase, based on inspection of the crystal structure, followed by growth-based screening, gives mutants that improve the growth of yeast on xylose. The mutations differ from those found earlier in random mutant libraries constructed by error-prone PCR. The new mutants described here and the two mutants discovered earlier, did not show improved xylose isomerization kinetics when tested in vitro with in the presence of an excess of single metals. Yet, metal occupation of the enzyme is of key importance as indicated by the low in vivo activities and the high calcium content found by metal analysis and X-ray crystallography of xylose isomerase isolated from yeast. Small differences in relative metal affinities and activities can explain the improved growth caused by mutations in the second shell of metal coordination. Rational redesign of xylose isomerase for better in vivo performance would require a theoretical framework that describes xylose isomerase structure–activity relations as a function of metal incorporation and activation.

## Methods

### Strain and plasmid construct

The *E. coli* strain used in this work is NEB 10 β (New England Biolab). *S. cerevisiae* strain DS75543 is derived from RWB217 [16] and was constructed for xylose fermentation by genetic engineering and further improved for growth rate on xylose by laboratory evolution. Plasmids bearing the *XKS1* and *Pir*XI expression cassette were cured from the strain, and an *XKS1* overexpression cassette was re-introduced by integration in the yeast genome. The relevant genotype of DS75543 is the following: MATa, *ura3*-*52*, *leu2*-*112*, *gre3::lox*P, *lox*P-P*tpi*::TAL1, *lox*P-P*tpi::RKI1*, *lox*P-P*tpi::TKL1*, *lox*P-P*tpi::RPE1*, *TY1::*P*adh1XKS1 *+ *LEU2*.

A yeast codon-optimized xylose isomerase gene-expression cassette, P_*TPI1*__*XylA*_T_*CYC1*_, was obtained from Prof. J.T. Pronk, TU Delft, and cloned into the 2µ plasmid pRS426-URA using SacI and SalI restriction sites. For *E. coli* expression, a pBAD/myc-His-derived plasmid containing *E. coli* codon-optimized *XylA* was used as described in our previous study [22].

### Library construction

**Fig. 11:**
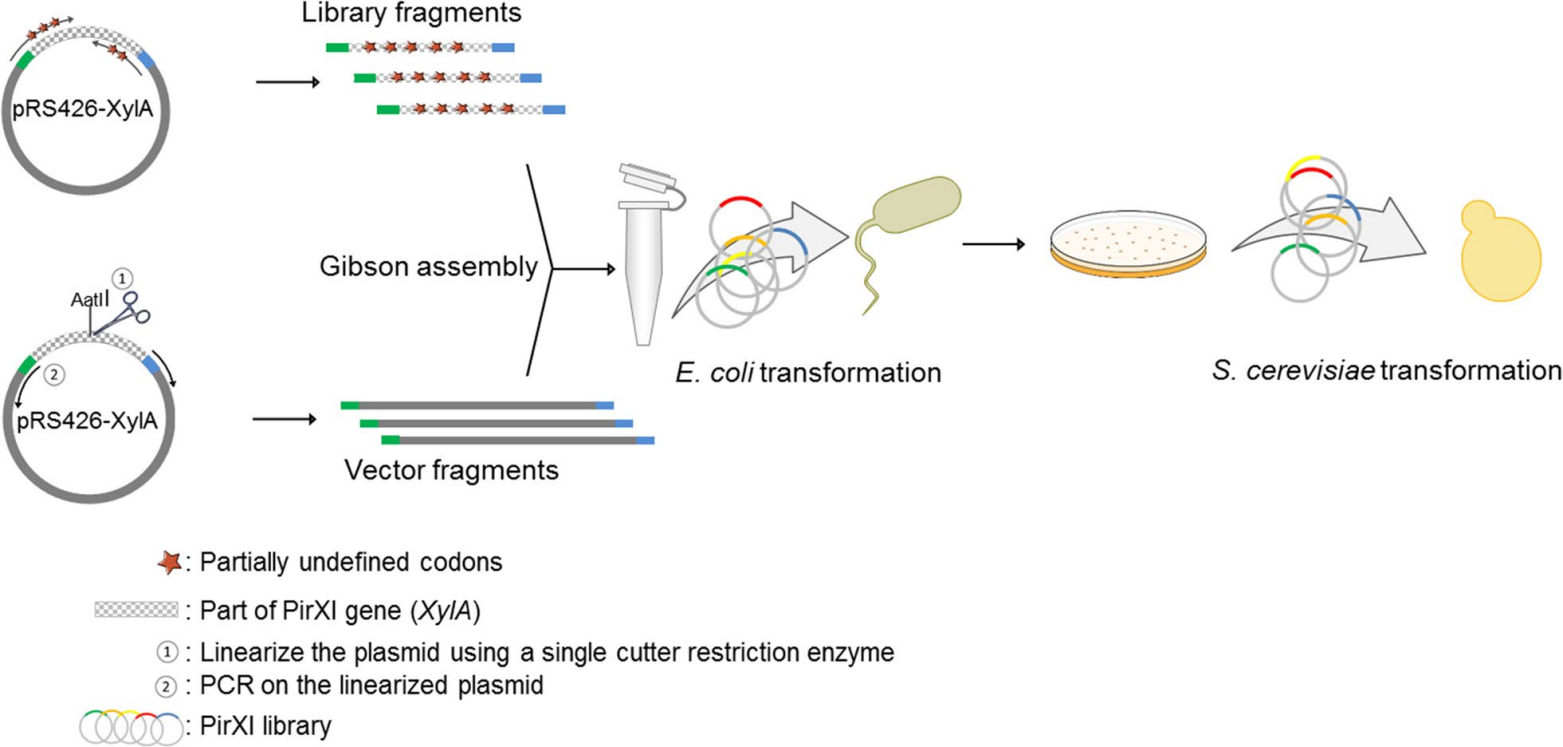
Library construction scheme

The library construction strategy is schematically shown in Fig. 11. A mutant PirXI library was created by cloning *XylA* fragments containing mutations into the pRS426-URA vector. The fragments and the vector were designed to contain > 20 bp overlaps for use of the Gibson assembly method for cloning. Appropriate partially undefined codons for generating the library were designed using a spreadsheet-based site-restricted library design tool called CoFinder [33]. Using primers containing the partially undefined codons and pRS426_pTPI_XylA as the template, PCR was performed to generate the library fragments. For creating the backbone, linearized pRS426_pTPI_XylA was used as the PCR template. The plasmid was linearized by using a restriction enzyme that cuts the DNA once outside of the backbone region. An AatII site was created for library LibM1 and the single existing BglII site was used for library LibM2. For both the fragment and the backbone generation, Phusion high-fidelity polymerase (ThermoFisher) was used and the instructions of the manufacturer were followed for the PCR reactions. After PCR, template fragments were degraded by incubating the reaction mixtures with 1 µl of DpnI at 37 °C for 3 h. For Gibson assembly of library fragments and backbone, a total of ~ 100 ng DNA was added at a 1:3 ratio of purified backbone to gene fragments. The library DNA fragments were mixed proportionally to the number of variants that is theoretically carried by each fragment. The assembly reactions were performed following the protocol established by Gibson et al. [56]. Subsequently, three aliquots of 100 µl *E. coli* were transformed with 10 µl reaction mixtures and transformants were selected on LB agar plates containing 50 µg ml^−1^ ampicillin. Plasmids from the entire *E. coli* transformant mixture were isolated, sequenced to confirm the diversity of the library and transformed to *S. cerevisiae*.

### In silico prediction of enzyme stability

The relative folding free energy differences ΔΔ*G*^Fold^ between PirXI wild type and variants were predicted using FoldX calculations [32] based on the X-ray structure of the wild-type enzyme (5NH7) and in silico-generated mutants.

### Site-directed mutagenesis

Selected PirXI variants for expression in *E. coli* and *S. cerevisiae* were reconstructed by QuikChange site-directed mutagenesis. For PCR, PfuUltra II Hotstart master mix (Agilent) was used following the manufacturer’s instructions. Subsequently, 1 µl DpnI was added to the reaction mixture, which was incubated at 37 °C for 3 h. Next, 10 µl of the reaction mixture were used to transform 100 µl NEB 10 β cells.

### Transformation

For *E. coli* transformation RhCl_2_-competent NEB 10 β cells were used and a standard heat-shock protocol was applied. *S. cerevisiae* transformation was performed using the LiAc/SS carrier-DNA/PEG method. The protocol established by Gietz et al. was followed [57], adjusting the duration of heat-shock to that works best for the DS75543 strain which was approximately 1 h.

### Yeast cultivation

For all yeast growth experiments a defined medium prepared according to Verdyun et al. [58] was used, either supplemented with 20 g l^−1^ glucose (glucose medium) or 20 g l^−1^ xylose (xylose medium) unless stated otherwise. For solid medium 2% agar was added prior to autoclaving. Cells were pre-grown on glucose medium and leftover glucose was washed away with ddH_2_O. The washed cells were resuspended in xylose medium and diluted appropriately. On-line growth measurements of *S. cerevisiae* were performed using a microplate reader (Synergy H1, BioTek). For this, 200 µl xylose medium was used in 96-well cell culture plates (Eppendorf) and samples from pre-cultures were added to an OD_600_ of 0.02. Plates were covered with an optical-clear gas-permeable seal (Breath-easy, Diversified Biotech). Cultures were grown at 30 °C with continuous shaking (linear, 731 pm, 2 mm shaking amplitude) and the OD_600_ was measured with 30-min intervals. For off-line growth measurements cells were cultivated at 30 °C in 100-ml shake flasks containing 25 ml xylose medium and the optical densities at 600 nm was followed in a spectrophotometer using plastic cuvettes.

### Library screening by competitive growth

After transformation, yeast cells were plated on solid glucose medium. All transformants were collected by gently scraping the colonies from the plate and divided for duplicates of aerobic and anaerobic screening. Prior to the screening, cells were pre-grown on glucose until the mid-exponential phase. For the aerobic screening, pre-cultures prepared were diluted in 50 ml xylose medium to an OD_600_ of ~ 0.1 and grown at 30 °C with shaking at 135 rpm. For anaerobic growth, cells prepared from pre-cultures were inoculated in 100 ml xylose medium supplemented with 420 mg l^−1^ Tween 80 and 10 mg l^−1^ ergosterol. To keep the conditions oxygen-limited, the cultures were grown in 100-ml Schott glass bottles which were kept air-tight with a rubber stopper and a glass airlock. Autoclaved medium was flushed with argon for at least 20 min prior to inoculation. The cultures were grown at 30 °C without shaking, but occasionally stirred briefly to keep homogenous cell suspensions. For aerobic and anaerobic screening, transfers to fresh xylose medium were carried out when the cultures reached an OD_600_ of 8–10 and 2, respectively. After the 10th transfer, cultures were harvested to investigate the evolved library diversity. Cells were pre-treated with zymolyase (Amsbio) and plasmids were isolated using a miniprep kit (Qiagen). The isolated plasmids were transformed to *E. coli* to produce sufficient plasmid DNA for sequencing. The plasmids were isolated from *E. coli* transformants using a miniprep kit and sequenced by GATC (Konstanz, Germany).

### Library screening by comparative growth

All library transformants were collected from glucose medium plates, washed and diluted with sterile ddH_2_O and plated on solid xylose medium. After 2–3 days of incubation, around 100 colonies that grew faster were selected by comparing colony sizes with cells expressing wild-type PirXI. Plasmids were isolated from selected transformants as described above and retransformed into fresh non-evolved yeast cells. The selection procedure was repeated twice and after the last round of yeast transformation and selection approximately 200 random colonies were picked and replicated both on glucose and xylose plates. Several colonies harboring wild-type PirXI were included as controls. Next, 46 colonies that grew faster than the controls on the xylose medium were selected and the corresponding colonies were picked from the glucose plate. The growth of the selected transformants in liquid xylose medium was measured in a 96-well plate using a microtiter plate reader. The growth experiments with these 46 transformants and wild-type PirXI carrying clones were performed in duplicate. The 24 best growing colonies were selected, plasmids were isolated and sequenced as described above.

### Enzyme expression in *E. coli* and purification

For in vitro enzyme analysis, PirXI variants were expressed in *E. coli* and purified. NEB 10β cells harboring PirXI variants were grown in TB medium (12 g/l tryptone, 24 g/l yeast extract, 5 ml/l glycerol, 2.31 g/l KH_2_PO_4_, and 16.43 g/l K_2_HPO_4_·3H_2_O) containing 50 μg ml^−1^ ampicillin at 37 °C. For inducing expression, 0.2% (w/v) l-arabinose was added and the cells were cultivated for 16 h at 37 °C. The cells were harvested by centrifugation and purification of overexpressed XI was done as described in previously [22].

### In vitro enzyme activity and metal affinity

For measuring the XI activity in the presence of different metal cofactors, purified enzymes were first incubated overnight with 10 mM EDTA. Subsequently, any metal–EDTA complex and excess EDTA were removed by buffer exchange to 20 mM MOPS (pH 7.0) using EconoPac 10-DG desalting columns (Bio-Rad). All enzyme activities were measured with sorbitol dehydrogenase (SDH, Roche Diagnostics GmbH)—coupled assay at 30 °C and pH 7.0 (20 mM MOPS). The reactions were followed either using a spectrophotometer (1 ml mixtures) (Jasco) or with a microplate reader in (200 µl reactions) (Synergy H1, BioTek). All reactions contained 0.15 mM NADH, 1 mM divalent metal, 1.5 unit/ml SDH and d-xylose. The mixtures were incubated at 30 °C for 5 min and the reactions were initiated by addition of 0.05–0.2 µM apo XI. For measuring the activation constant for each metal, activities of XI on 100 mM (for Mg^2+^ and Mn^2+^) or 400 mM (for Ca^2+^) d-xylose in the presence of various concentrations of metals were determined using a microplate reader. Various concentrations of metals and PirXI were used according to the level of metal-dependent activity of the enzyme.

### Thermostability measurement

Thermostability of PirXI variants were determined by measuring an increase in fluorescence of Sypro Orange (Life Technologies, Carlsbad, CA, USA) during thermal unfolding [59]. The change of fluorescence emission at 575 nm was measured with CFX RT-PCR system (Biorad) while increasing the temperature from 20 to 90 °C at a rate of 0.5 °C min^−1^. In order to evaluate the metal affinity of PirXI variants, various concentrations of divalent metal ions (MgCl_2_, MnCl_2_ or CaCl_2_) ranging from 0 to 1.28 mM were included in 25-µl reaction mixtures containing 1 mg/ml apo PirXI and 2× of Sypro Orange dye.

### Cell extract activity

*Saccharomyces cerevisiae* cells grown on xylose were harvested, washed with sterile ddH_2_O and resuspended with lysis buffer containing 20 mM MOPS (pH 7.0) and 100 U/g cells (w/w) zymolyase (Amsbio). The cell suspension was incubated at 30 °C with mild shaking at 50 rpm for 20 min. Next, the zymolyase-treated cells were disrupted by vortexing for 30 s with an equal volume of glass beads followed by cooling on ice for 1 min. The process was repeated five times. The cell lysate was spun down by centrifugation at 17,000×*g* and the supernatant was collected as the cell-free extract (CFE). The total protein concentration of the CFE was measured by the Bradford assay using bovine serum albumin (BSA) as the standard. Activity of the CFE on 100 mM xylose was measured by the SDH-coupled assay as described above without addition of any metals.

### Enzyme expression levels

Crude extracts of *S. cerevisiae* expressing either wild-type or V270A–A273G PirXI grown on xylose were prepared by lysing the cells as described above. Total protein concentrations were determined by the Bradford assay and 2 µg of protein from each cell extract was used for SDS-PAGE analysis. Along with the crude extract samples, 0.2, 0.4, 0.6, 0.8, 1 and 2 µg of purified PirXI were loaded on the same gel as references. An image of the gel was taken and analyzed using the image processing program Image J (https://imagej.net). The intensity of the bands corresponding to PirXI was quantified and the expression levels were calculated.

### Purification of PirXI expressed in *S. cerevisiae* for crystallography

A single colony of DS75543 cells containing PirXI was inoculated in 5 ml glucose medium and grown overnight. Next, cultures were diluted into a 50 ml glucose medium to an OD_600_ of 0.1 and cultivated at 30 °C until mid-exponential phase. The cells were collected by centrifugation, washed and diluted into a 2 l xylose medium to a starting OD_600_ of 0.1. The cultures were grown at 30 °C and harvested when the OD_600_ reached 3–4. In order to minimize metal contamination and preserve in vivo enzyme-bound metals, we applied a gentle cell lysis method and a minimal purification step required as described below. Buffers used are as follows: A, 10 mM MOPS, pH 7.5; B, 10 mM MOPS, pH 7.5 + 5 mM DTT; C, 10 mM MOPS, pH 7.5 + 0.5 M sucrose; D, 10 mM MOPS, pH 7.5 + 0.5 M sucrose + 100 U/g cells (w.w.) of zymolyase + EDTA-free protease inhibitor cocktail tablets (Roche); and E, 10 mM MOPS, pH 7.5 + 0.5 M KCl. The harvested cells were washed with buffer A and the pellets were resuspended in buffer B and subsequently incubated for 10 min on ice. The cells were centrifuged and washed with buffer C. The pellets were resuspended in 25 ml of buffer D and incubated at 30° while gently shaken at 50 rpm for 1 h. The disruption of the cell walls of the yeast was monitored under a light microscope. Next, 25 ml of buffer A was added to the cell lysate mixtures and the cells were spun down at 1500 g for 5 min. The supernatants were carefully collected and centrifuged at 16,500 rpm for 30 min. PirXIs were purified from the CFE using an anion exchange column (Resource Q) by applying an ionic strength gradient using buffer A and buffer E. Prior to sample loading the CFEs were diluted appropriately to reduce the ionic strength of the samples to a similar level of buffer A. Most of PirXI eluted with 50–100 mM KCl and the fractions of highest purity were collected for crystallization.

### Crystallization and structure determination

Wild-type xylose isomerase and XI mutant V270A–A273G were crystallized by the hanging-drop vapor diffusion method with 14–17% PEG3350, 0.08 M ammonium sulfate and 0.1 M HEPES, pH 7.0 [22]. For soaking experiments, the stabilizing solution was supplemented with 2 M xylose. Datasets were collected at the in-house source at 110 K [22]. Details and processing statistics are given in Table 4. Processing was done with XDS [60]. The structure of xylose isomerase (PDB code 5NH5), with all waters and ligands removed, was used as a starting model. Refinement was done with Refmac5 [61]. Sugar ligands and ions were manually placed in sigmaA-weighted 2Fo–Fc, Fo–Fc and anomalous electron density maps [62] with the program Coot [63]. The atomic coordinates and the structure factors of the structures have been deposited in the Protein Data Bank (code 6T8E for native and 6T8F for V270A–A273G PirXI.

**Table 4:** Data collection and refinement statistics

	Wild type	V270A–A273G
Resolution range (Å)^a^	46.6–1.86 (1.89–1.86)	46.6–2.0 (2.03–2.00)
Cell dimensions		
*a*, *b*, *c* (Å)	78.5, 79.3, 91.9	78.6, 79.4, 92.0
*α*, *β*, γ (°)	115.5, 90.0, 117.1	115.5, 90.0, 117.1
Number of unique reflections	135,735 (5669)	111,563 (5325)
Completeness (%)	93.6 (79.1)	95.3 (92.1)
Overall *I*/*σ* (I)	7.1 (1.9)	5.3 (2.0)
*R*_merge_ (%)	6.7 (31.8)	6.8 (24.4)
*R/R*_free_ (%)	13.9/17.6	15.4/18.5
R.m.s. deviations from ideal values		
Bond lengths (Å)	0.009	0.010
Bond angles (°)	1.55	1.55
Protein residues		
Ca^2+^ ions	4 × 2	4 × 2
Water molecules	1907	1745
Xylose molecules	22	18
Sulfate ions	7	6
PDB accession ID	6T8E	6T8F

^a^Values in parentheses are for the highest resolution shell

## Data Availability

*E. coli* expression clones of PirXI mutants reported in this study are available from the corresponding author.

## Abbreviations

XI: xylose isomerase
PirXI: xylose isomerase from *Piromyces*
CFE: cell-free extract
SDH: sorbitol dehydrogenase
BSA: bovine serum albumin
